# Metabolic reprogramming of CD4⁺ T cells by Zaprinast induces HIV-1 latency reversal *ex vivo*

**DOI:** 10.1186/s12977-026-00680-x

**Published:** 2026-06-13

**Authors:** V. De Masson d’Autume, V. LeDouce, A. Dutilleul, S. O’Reilly, N. François, E. Formiconi, M. Angieladis, L. Pilosio, M. Bendoumou, A. Garcia Leon, D. Alalwan, E. Moore, T. McGinty, J. C. Valle-Casuso, A. Macken, O. Rohr, C. Van Lint, A. Sáez-Cirión, A. Cotter, E. Feeney, P. W. G. Mallon, W. V. Gautier

**Affiliations:** 1 UCD Centre for Experimental Pathogen Host Research (CEPHR), University College Dublin, Belfield, Dublin 4, Ireland; 2 School of Medicine, University College Dublin, Belfield, Dublin, Ireland; 3 Department of Infectious Diseases, Mater Misericordiae University Hospital, Eccles St, Dublin 7, Ireland; 4 Department of Infectious Diseases, St Vincent’s University Hospital, Elm Park, Dublin 4, Ireland; 5 Institut Pasteur, Viral Reservoirs and Immune Control Unit, Université Paris Cité, Paris, France; 6 Université de Strasbourg, UPR 9002 CNRS, IUT Louis Pasteur, Schiltigheim, France; 7 Service of Molecular Virology, Department of Molecular Biology (DBM), Université Libre de Bruxelles (ULB), 6041 Gosselies, Belgium; 8 Conway Institute of Biomedical and Biomolecular Research, University College Dublin, Belfield, Dublin 4, Ireland

## Abstract

**Background:**

HIV-1 latency and persistence of viral reservoirs within memory CD4^+^ T cells remain a fundamental obstacle to achieving a cure despite suppressive antiviral treatments. HIV-1 persistence is sustained by the dynamic interactions between viral regulatory mechanisms and the host cellular environment. At the intersection between immunometabolism and virology, the quiescent metabolic profile of resting CD4^+^ T cells, defined as the balance between oxidative phosphorylation (OXPHOS) and aerobic glycolysis, supports the long-term maintenance of latent viral reservoirs. Existing “Shock and Kill” strategies have shown limited clinical impact, partly due to the metabolic constraints that limit robust viral reactivation. Targeting metabolic junctions to overcome this barrier may provide a complementary therapeutic avenue.

**Results:**

We evaluated Zaprinast, a mitochondrial pyruvate carrier inhibitor (MPCi), for its capacity to reprogramme CD4^+^ T cell metabolism and promote latency reversal. Across multiple primary T-cell based models of HIV-1 latency, Zaprinast induced a moderate yet reproducible increase in HIV-1 gene expression and viral particle production, including in circulating reservoirs from antiretroviral-treated individuals cultured *ex vivo*. Metabolic profiling revealed a biphasic response: an initial, transient inhibition of mitochondrial respiration followed by a shift from an OXPHOS-dominant to a more glycolytic metabolic state, while maintaining mitochondrial function. This metabolic reprogramming of resting CD4^+^ T cells by Zaprinast was reversible and did not impair cell viability, trigger non-specific T cell activation or proliferation, nor elevate reactive oxygen species levels.

**Conclusions:**

These results highlight that selective targeting of the quiescent metabolic state in resting CD4^+^ T cells can facilitate HIV-1 reactivation without compromising cellular integrity. This study identifies host metabolic reprogramming as a promising strategy to enhance latency reversal and complement existing cure strategies. Our work provides new insights into the importance of host metabolic states in governing viral persistence and underscores the translational potential of metabolic interventions in HIV-1 eradication research.

**Supplementary Information:**

The online version contains supplementary material available at 10.1186/s12977-026-00680-x.

## Introduction

Despite suppressive antiretroviral therapy (ART), HIV-1 persistence in latent reservoirs within memory CD4^+^ T cells remains the primary obstacle to achieving a cure [1–4]. Current “Shock and Kill” strategies, which aim to force HIV-1 latency reversal for immune-mediated clearance while maintaining ART, have shown limited clinical efficacy [1, 5, 6]. While these approaches target epigenetic and transcriptional blocks mediating HIV-1 gene silencing, they insufficiently address the specific metabolic context that underpins viral reservoirs persistence [7–9]. Recent research focusing on CD4^+^ T cell metabolic plasticity during HIV-1 replication highlights the critical roles of aerobic glycolysis and OXPHOS on two distinct phases of the viral lifecycle [8, 10, 11]. During active replication, HIV-1 preferentially targets glycolytically active CD4^+^ T cell subsets while also driving T cell metabolic reprogramming to promote aerobic glycolysis, pentose phosphate pathways (PPP), and OXPHOS to fuel reverse transcription, integration, gene expression, and viral biosynthesis [11–16]. This metabolic remodelling parallels the Warburg effect observed in cancer cells, where glycolytic flux is prioritised despite oxygen availability to support biosynthetic demands [17]. The heightened metabolic activity of infected cells is sustained by increased glucose and amino acid (e.g. glutamine) uptake, and lipid metabolism [18]. At the molecular level, mTOR acts as a central orchestrator, promoting glycolysis, PPP activity, and glutaminolysis, while viral regulatory proteins co-opt metabolic enzymes and signalling pathways, facilitating this metabolic re-wiring to create a pro-viral environment [10, 19–25]. Conversely, the establishment and persistence of HIV-1 latent reservoirs are intricately linked to the quiescent metabolism of CD4^+^ memory T cells reliant on OXPHOS rather than aerobic glycolysis to sustain their basal needs, long-term survival, and their homeostatic proliferation [14, 26]. This distinct metabolic profile plays a significant role in facilitating HIV-1 silencing and the long-term persistence of viral reservoirs [27, 28]. Recent evidence even suggests that HIV infection reprogrammes CD4^+^ T cell metabolism towards quiescence, driving HIV into latency [29, 30]. Additionally, CD8^+^ T cells also play a key role by reinforcing the quiescent metabolic state of infected CD4^+^ T cells and promoting the establishment of HIV-1 latency and the persistence of viral reservoirs [31, 32]. The importance of addressing metabolic restrictions when developing HIV-1 cure strategies is supported by clinical trials showing that mTOR inhibition reduced cell-associated (CA) HIV-RNA levels in circulating CD4^+^ T cells from ART-suppressed People Living With HIV-1 (PLWH) [33–35]. Nevertheless, this metabolic duality presents a critical challenge as HIV-1 latency reversal from inducible reservoirs requires both the restoration of glycolytic activity while maintaining OXPHOS [15, 27].

The central role of immunometabolism in regulating HIV-1 latency and reactivation prompted us to investigate the regulatory networks at the nexus between glycolysis and mitochondrial respiration. We identified the cytoplasmic pool of pyruvate, end product of glycolysis, as a key metabolic junction linking aerobic glycolysis, mitochondrial tricarboxylic acid (TCA) cycle and OXPHOS [36]. When pyruvate is converted to lactate, it promotes aerobic glycolysis, whereas its mitochondrial import fuels the TCA cycle and supports OXPHOS (Fig. 4) [37–39]. Notably, previous studies have shown that the intracellular fate of pyruvate can be controlled by inhibiting the Mitochondrial Pyruvate Carrier (MPC). Restricting pyruvate entry into mitochondria diverts it towards lactate production, thereby shifting cellular metabolism to a more glycolytic state [40]. Zaprinast has been characterised as a potent MPCi with an IC_50_ of 321 nM and has been tested across multiple cell types, including primary murine retina cells, hepatocyte cell lines, and adipocytes, but not yet assessed in primary CD4 T cells [37, 38, 41–43]. Functionally, Zaprinast-mediated inhibition of pyruvate mitochondrial transport reprogrammes cellular metabolism by reducing mitochondrial respiration and/or promoting glycolytic flux [37, 38, 40, 44–46].

In this study, we evaluated Zaprinast potential to reverse HIV latency *ex vivo* in inducible HIV-1 reservoirs from ART-suppressed individuals and its effect on CD4^+^ T cell metabolic reprogramming. Our results indicate that targeting the quiescent metabolic state of CD4^+^ T cells represents a promising avenue to enhance “Shock and Kill” therapeutic strategies.

## Results

### The mitochondrial pyruvate carrier inhibitor Zaprinast reactivates HIV-1 in vitro

**Fig. 1:**
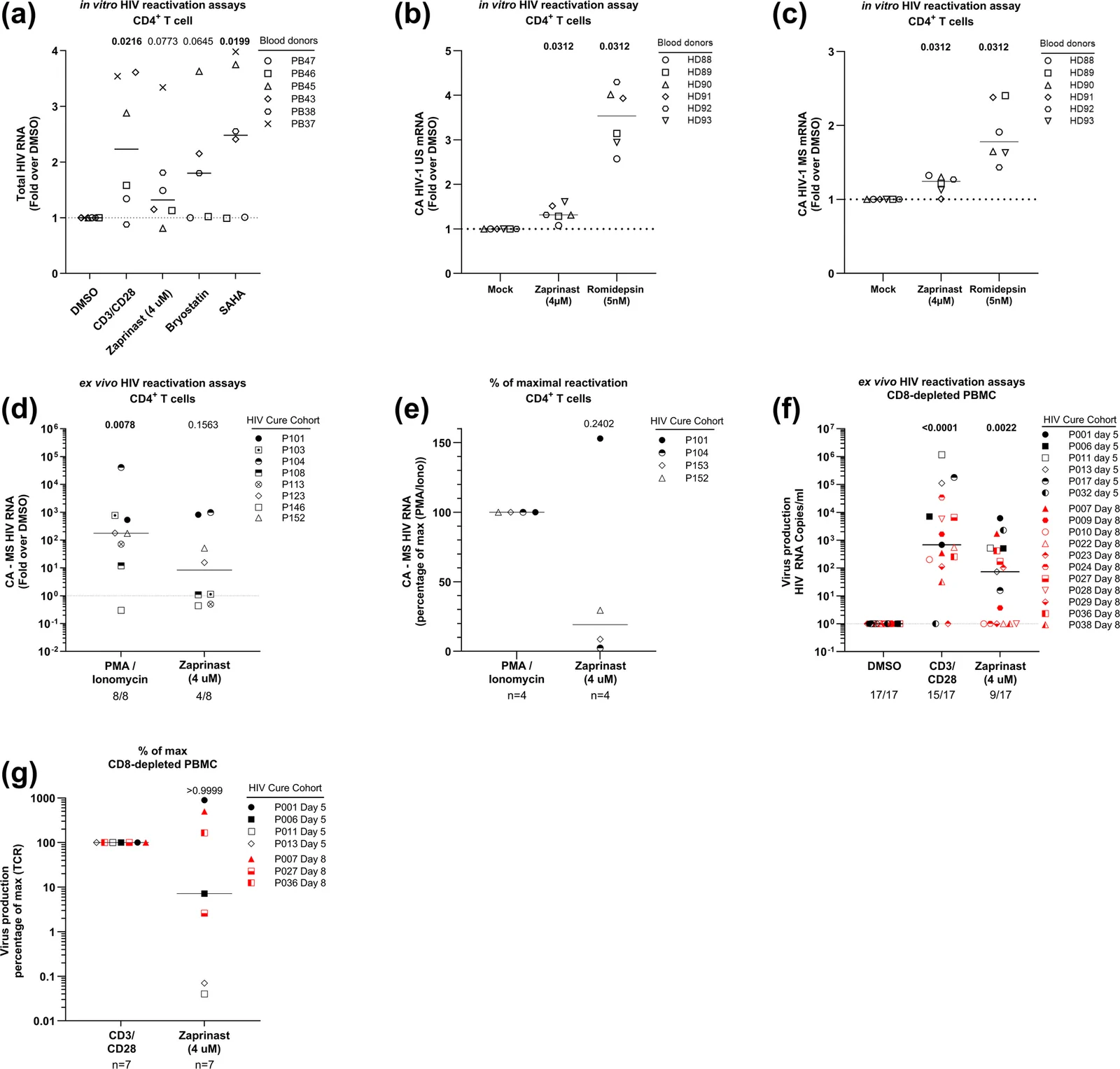
Zaprinast induces HIV-1 latency reversal in vitro and *ex vivo*. **a** Total HIV-1 RNA expression in primary CD4^+^ T cells latently infected with HIV-1 in vitro after a 24-hour treatment. Cells were treated with DMSO (vehicle control), Zaprinast, bryostatin, SAHA or anti-CD3/anti-CD28 treatments (n = 6). Data are shown as relative quantifications. Statistical analysis was performed using paired t-test versus DMSO. **b** Cell-associated (CA) Unspliced (US) HIV-1 RNA expression in CD4^+^ T cells infected in vitro with HIV-1 VSV-G pseudotyped particles after 72 h of treatment with DMSO (vehicle control), Zaprinast (4 µM) or Romidepsin (5nM) (n = 6). Data are shown as relative quantification. Statistical analysis was performed using Wilcoxon test versus DMSO. **c** CA Multispliced (ms) HIV-1 RNA expression in CD4^+^ T cells infected in vitro with HIV-1 VSV-G pseudotyped particles after 72 h of treatment with DMSO (vehicle control), Zaprinast (4 µM) or Romidepsin (5nM) (n = 6). Data are shown as relative quantification. Statistical analysis was performed using Wilcoxon test versus DMSO. **d** CA HIV-1 MS RNA expression in CD4^+^ T cells from ART-suppressed individuals after 72 h of treatment. Cells were treated with DMSO (vehicle control), Zaprinast or PMA/Ionomycin (n = 8). Data are shown as relative quantifications. Statistical analysis was performed using Wilcoxon test versus DMSO. **e** CA HIV-1 MS RNA expression as a percentage of the maximal HIV-1 reactivation induced by PMA/Ionomycin treatment. Data are shown for 4 samples exhibiting HIV-1 reactivation in both treatments. Statistical analysis was performed using paired t-test versus DMSO. **f **HIV-1 virus production in CD8-depleted PBMCs from ART-suppressed individuals on day 5 (black symbols) or day 8 (red symbols) post-stimulation. Cells were treated with DMSO, anti-CD3/anti-CD28 antibodies or Zaprinast (n = 17). Data are shown as absolute quantifications. Statistical analysis was performed using paired t-test versus DMSO. **g** Virus production expressed as a percentage of TCR stimulation for samples exhibiting HIV-1 reactivation in both treatments (n = 7). Statistical analysis was performed using paired t-test versus DMSO. Median for each condition are displayed with a dark line.

We identified Zaprinast, an MPCi, as a molecule of interest for latency reversal in quiescent HIV-1 cellular reservoirs, where metabolic activity remains insufficient to support robust viral replication. To evaluate its latency reversal capacity, we first employed an in vitro primary cellular model of HIV-1 latency adapted from the Planelles model [47]. It enables activated, replication-competent HIV-1-infected primary CD4^+^ T cells to transition toward a memory phenotype closely mimicking the quiescent reservoir environment observed in vivo [47]. Viral reactivation was quantified at the transcriptional level by measuring total viral transcripts after 18 h of treatment with TCR stimulation, DMSO (negative control) or drugs of interest, including Zaprinast, the HDAC inhibitor SAHA and the PKC agonist Bryostatin, two well-characterised Latency Reversing Agents (LRAs) [48–50]. Zaprinast (4 µM) induced a moderate and consistent increase in HIV-1 gene expression, with levels of reactivation that were generally lower than, yet within the range of inter-donor variability observed for pan-TCR stimulation and the two established LRAs (Fig. 1a).

### Zaprinast induces HIV-1 US RNA and MS RNA expression in CD4^+^ T cells infected in vitro

To further assess the impact of Zaprinast on cell-associated HIV-1 transcription, we used a primary infection model in which resting CD4^+^ T cells from blood donors were infected in vitro with VSVg-pseudotyped HIV-1 particles and treated for 72 h with DMSO (negative control), Zaprinast (4 µM), or the HDAC inhibitor Romidepsin (5 nM) [9, 51]. We quantified intracellular HIV-1 unspliced (US) mRNA and multi-spliced (MS; tat/rev) RNA species. Across six independent donors, Zaprinast consistently increased cell-associated HIV-1 RNA, with a mean 1.35-fold fold induction of US RNA (*p* = 0.0312) and 1.2-fold induction of MS RNA (*p* = 0.0312) (Fig. 1b and c). In the same samples, Romidepsin induced higher levels of HIV-1 transcriptional reactivation, with an average 3.48-fold increase in US RNA (*p* = 0.0312), and 1.9-fold increase of MS RNA (*p* = 0.0312) consistent with its established potency as an LRA [52].

### Zaprinast induces HIV-1 MS RNA expression in CD4^+^ T cell reservoirs from ART-suppressed individuals

To evaluate the efficacy of Zaprinast as an LRA, we further examined its capacity to induce HIV-1 gene expression from *bona fide* circulating viral reservoirs cultured *ex vivo.* CD4^+^ T cells isolated from ART-treated and virally suppressed PLWH (Table 1) were cultured ex vivo for 72 h with DMSO (negative control), PMA/Ionomycin (positive control for T cell activation) or Zaprinast (4 µM), and intracellular levels of HIV-1 MS RNA levels were quantified. Tat-Rev MS RNA levels serve as a critical biomarker for productive HIV-1 reactivation and have been shown to predict virion production both ex vivo and in vivo [53–57]. Among the eight samples with PMA/Ionomycin inducible HIV-1 cellular reservoirs, Zaprinast induced HIV-1 MS RNA expression in four donors, with fold-changes ranging from 16-fold to 983-fold over DMSO (*p* = 0.1563) (Fig. 1d). Restricting the analysis to these 4 responders, Zaprinast achieved on average19.13% of the maximal reactivation observed with PMA/Ionomycin, highlighting a moderate but consistent latency-reversing activity ex vivo in these circulating HIV-1 reservoirs (Fig. 1e). No significant correlation was observed between proviral load and the level of HIV reactivation measured (Table 1, Data not shown).

**Table 1:** Study participant characteristics

Study participants characteristics	Ex vivo CD4^+^ T cells	Ex vivo CD8^+^-depleted PBMCs
	*N* = 21^*1*^	*N* = 17^*1*^
Age (Years)	39.6 (35.2, 41.7)	41.4 (37.5, 46.3)
Sex at birth		
F	3 / 21 (14%)	3 / 17 (18%)
M	18 / 21 (86%)	14 / 17 (82%)
CD4^+^ T cell count	625.5 (499.5, 765.5)	670.5 (468.0, 838.0)
Missing	1	1
CD4/CD8 ratio	1.1 (0.8, 1.6)	0.7 (0.6, 1.2)
Missing	1	1
ART started in	2019 (2018, 2021)	2015 (2013, 2016)
Missing	4	1
ART regimen		
Atripla	0 / 17 (0%)	6 / 16 (38%)
Biktarvy	4 / 17 (24%)	0 / 16 (0%)
Delistrigo (Doravirine, Lamivudine, Tenofovir)	1 / 17 (5.9%)	0 / 16 (0%)
Dolutegravir / Relipivirine	1 / 17 (5.9%)	0 / 16 (0%)
Dovato (Dolutegravir / lamivudine)	1 / 17 (5.9%)	0 / 16 (0%)
Elvitegravir, Cobicistat, Emitrictabine, TAF	1 / 17 (5.9%)	0 / 16 (0%)
Eviplera	2 / 17 (12%)	0 / 16 (0%)
Genvoya	0 / 17 (0%)	4 / 16 (25%)
Raltegravir_Darunavir_Ritonavir	0 / 17 (0%)	1 / 16 (6.3%)
Rezolsta_Dolutegravir	0 / 17 (0%)	1 / 16 (6.3%)
Striblid	1 / 17 (5.9%)	1 / 16 (6.3%)
TDF/FTC/ EFV	1 / 17 (5.9%)	0 / 16 (0%)
Triumeq	2 / 17 (12%)	2 / 16 (13%)
Truvada_Dolutegravir	3 / 17 (18%)	1 / 16 (6.3%)
Missing	4	1
NADIR	574.5 (359.0, 609.0)	127.5 (49.5, 482.0)
Missing	5	1
Integrated ProViral Load	69.9 (27.6, 174.3)	45.0 (4.0, 230.0)
Missing	8	0

^*1*^ Median (Q1, Q3); n / N (%)

### Zaprinast induces HIV-1 virion release from circulating reservoirs in CD8-depleted PBMC from ART-treated individuals

Having observed early reactivation events at transcriptional levels, our next objective was to determine whether Zaprinast could drive HIV-1 reactivation, ultimately promoting the completion of the viral replication cycle and the release of viral particles, a critical step for evaluating the potential of Zaprinast in HIV cure strategies. CD8-depleted peripheral blood mononuclear cells (PBMC) from 17 individuals on suppressive ART (Table 1) were cultured ex vivo with DMSO (negative control), Zaprinast (4 µM) or pan-TCR stimulation using anti-CD3/anti-CD28 antibodies for 5 or 8 days, depending on when viral reactivation was first detected. Viral particle production in cell-free supernatants was quantified by RT-qPCR following viral RNA extraction [58–60].

TCR stimulation-induced viral particle production in 15 out of 17 samples, with viral loads ranging from 32 to 1,166,546 copies/mL (*p* < 0.001) (Fig. 1f). Zaprinast induced viral particle production in 9 out of 17 samples, with levels of viral RNA ranging from 74 to 6,101 copies/mL (*p* = 0.0022) (Fig. 1f). Among the 17 samples, 2 samples were not reactivated under TCR stimulation but were reactivated under Zaprinast treatment, and 4 samples were reactivated under TCR stimulation and remained negative under Zaprinast treatment, underscoring the heterogeneity of reservoir responsiveness and the presence of distinct mechanistic blocks governing HIV-1 latency in different individuals [61]. Next, we compared the relative efficacy of Zaprinast with strong TCR stimulation in the 9 samples that produced virus under both conditions. Overall, Zaprinast achieved 7.13% of the maximal reactivation observed with pan-TCR stimulation (Fig. 1g). In this subset, Zaprinast exhibited a modest but significant latency reversing activity, supporting its ability to stimulate virion production from circulating HIV-1 reservoirs, although less efficiently than the pan-TCR-mediated T-cell activation.

### Zaprinast treatment does not induce cellular activation, proliferation or cytotoxicity

**Fig. 2:**
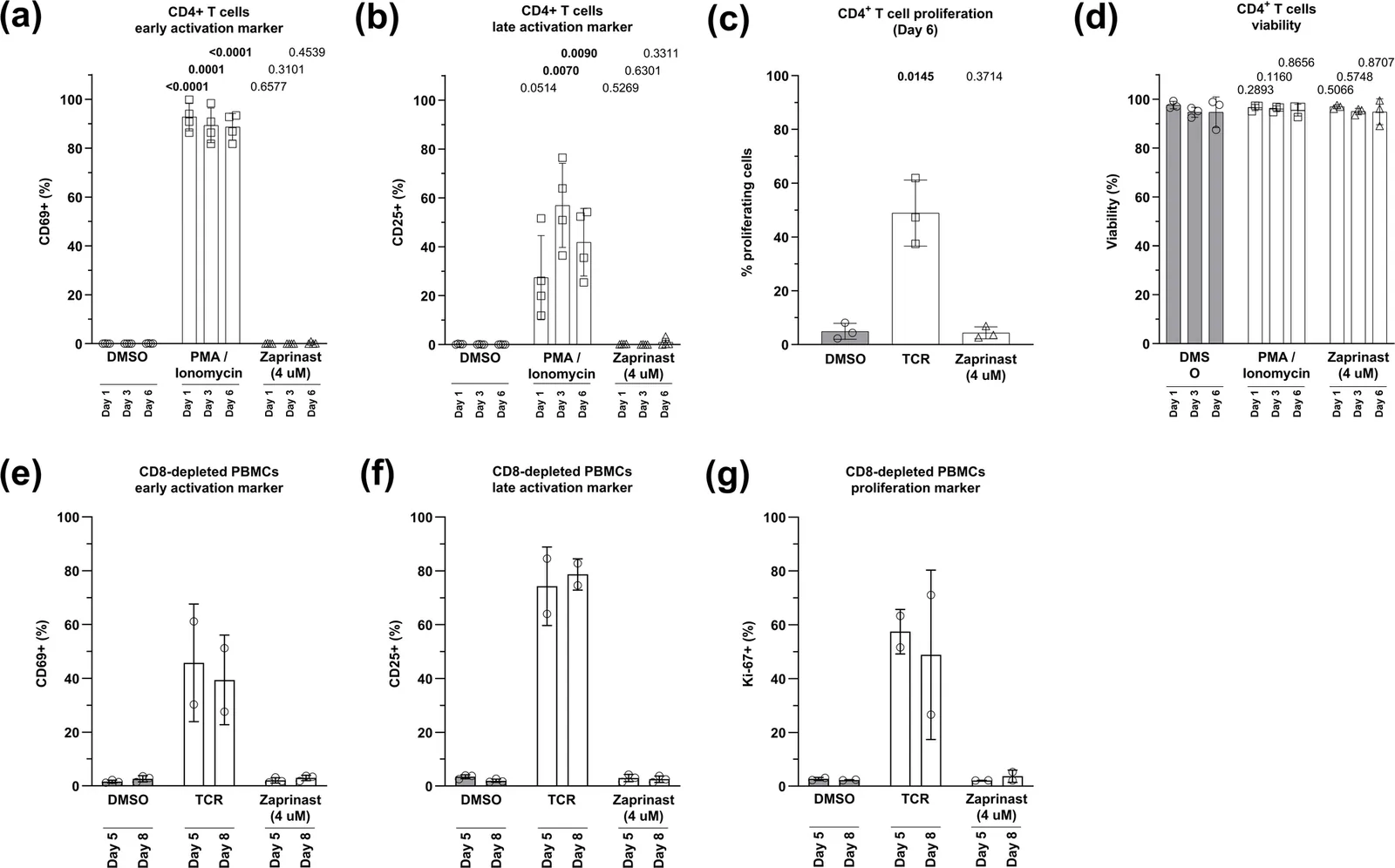
Zaprinast does not impact viability, activation, or proliferation of CD4^+^ T cells or CD8-depleted PBMCs. **a**, **b** Expression of activation markers CD69 (early) and CD25 (late) on primary CD4^+^ T cells from blood samples after 1, 3 and 6 days of treatment. Cells were treated with DMSO, 20 nM PMA and 280 nM Ionomycin (PMA/Ionomycin), or 4 µM Zaprinast (n = 3). Statistical analysis was performed using paired t-test versus DMSO. **c** Proliferation of primary CD4^+^ T cells from blood samples, assessed by CFSE dye after 1, 3 and 6 days of treatment. Cells were treated with DMSO, anti-CD3/CD28 antibodies (TCR activation), or 4 µM Zaprinast (n = 3). Statistical analysis was performed using paired t-test. **d** Viability of CD4^+^ T cells from blood samples using Viobility 405/452 Fixable Dye following 1, 3 and 6 days of treatment with DMSO, 20 nM PMA and 280 nM Ionomycin, or 4 µM Zaprinast (n = 3). Statistical analysis was performed using paired t-test versus DMSO. **e**, **f**, **g** Expression of activation markers CD69 (early) and CD25 (late) and cell proliferation using proliferation marker Ki-67 on CD8-depleted PBMCs from blood samples after 5 and 8 days of treatment. Cells were treated with DMSO, anti CD3/CD28 antibodies (TCR activation), or 4 µM Zaprinast (n = 2).

A critical consideration in the development of LRAs is their potential cytotoxicity and induction of global T-cell activation and proliferation, which could lead to adverse side effects. We evaluated the impact of Zaprinast (4 µM) on isolated CD4^+^ T cells and CD8-depleted PBMCs from blood donors by measuring cell viability, activation, and proliferation (Fig. 2). Zaprinast did not increase the frequency of cells expressing early (CD69) or late (CD25) activation markers, and did not reduce cellular viability up to 6 days in isolated CD4^+^ T cells and 8 days in CD8-depleted PBMCs (Fig. 2a, b, e and f). Critically, Zaprinast did not induce cellular proliferation as confirmed by CFSE dilution and Ki-67 staining (Fig. 2c and g). This absence of proliferative activity minimises the risk of clonal expansion of the HIV-1 reservoir, a safety advantage for potential latency reversing agents. This sharply contrasts with what was observed following stimulations with the global T cell activators PMA/Ionomycin or anti-CD3/anti-CD28 antibodies (Fig. 2).

### Zaprinast-mediated MCP inhibition exhibits a biphasic modulation of mitochondrial respiration in CD4^+^ T cells

Zaprinast, previously identified as a reversible MPC inhibitor, inhibits pyruvate uptake into the mitochondria, where it fuels the TCA, consequently reducing mitochondrial respiration [38, 44]. To investigate the impact of Zaprinast on basal primary CD4^+^ T cell metabolism, we conducted a series of real-time metabolic flux assays on live primary CD4^+^ T cells from blood donors.

**Fig. 3:**
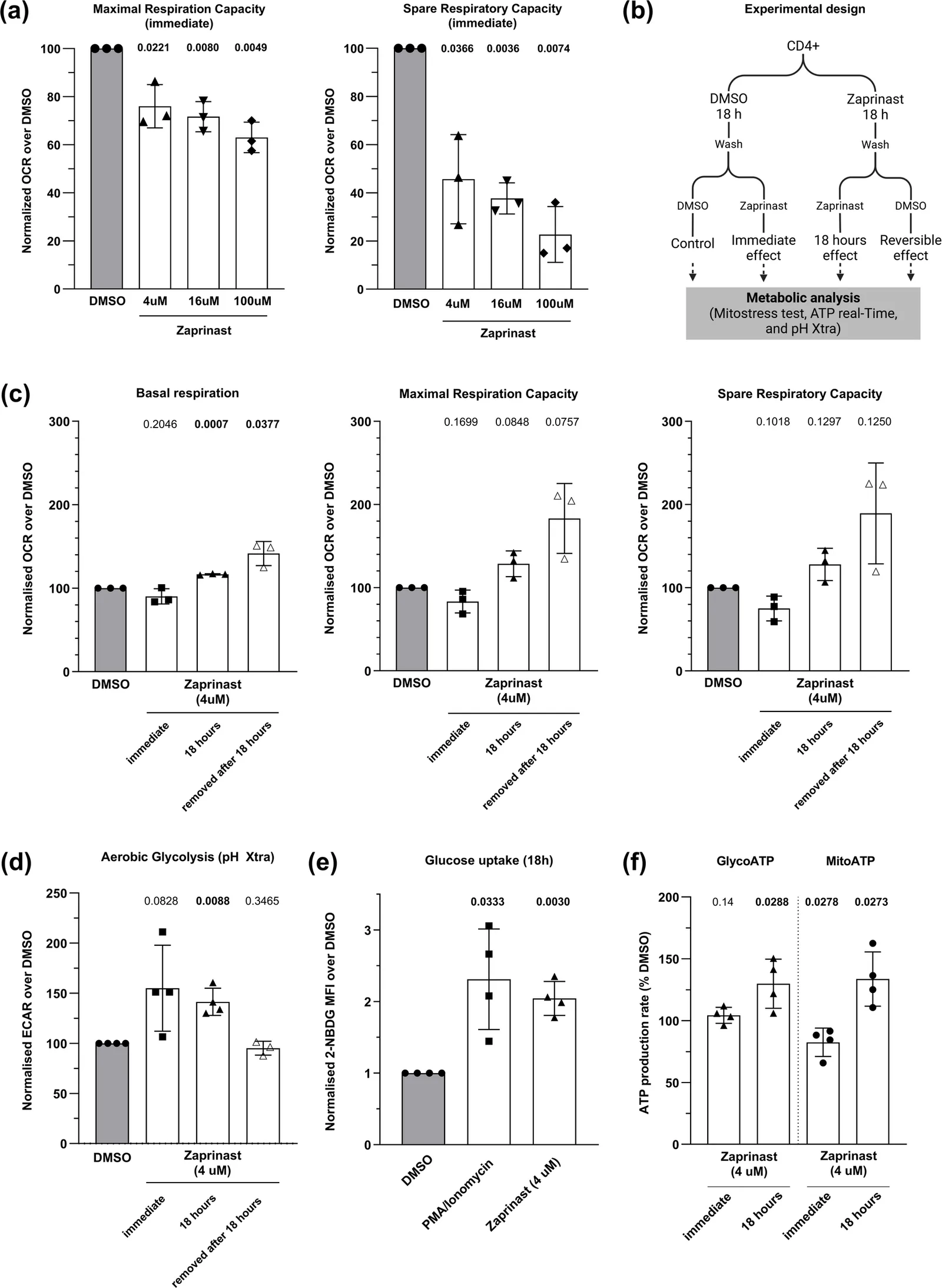
Zaprinast reprogrammes resting CD4^+^ T cell metabolism towards a more active state. **a** Immediate effects of Zaprinast on Maximal Respiratory Capacity and Spare Respiratory Capacity calculated from Oxygen Consumption Rate (OCR) measurements using Mitostress test (Agilent) on CD4^+^ T cells from PBMC from blood donors. Data shown from one representative experiment performed in triplicate. Statistical analysis performed using paired t-tests versus DMSO. **b** Experimental design of the following experiments **c** Maximal Respiratory Capacity, Spare Respiratory Capacity and Basal Respiration of primary CD4^+^ T cells calculated from OCR measurements using Mitostress test (Agilent) after immediate or 18-hour treatment with DMSO or 4 µM Zaprinast (n = 3). Statistical analysis was performed using paired t-tests versus DMSO. **d** Glucose uptake in primary CD4^+^ T cells after 18-hour treatment with 4 µM Zaprinast (n = 4), measured using glucose analogue 2-NBDG (Invitrogen). Statistical analysis was performed using paired t-tests versus DMSO. **e** Extracellular Acidification Rate (ECAR) of primary CD4^+^ T cells following immediate or 18 h treatment with DMSO or 4 µM Zaprinast treatment (n = 4), measured using pH Xtra assay (Agilent) with a microplate reader (Molecular Device ID3) over a 2-hour kinetics. Statistical analysis was performed using paired t-tests versus DMSO. **f** MitoATP and GlycoATP production in primary CD4+ T cells following immediate or 18 h treatment with 4 µM Zaprinast (n = 4), measured using ATP real-time assay (Seahorse Analyser, Agilent). Statistical analysis was performed using paired t-tests versus DMSO

We first examined the immediate impact of increasing Zaprinast concentrations on mitochondrial respiration. By measuring Oxygen Consumption Rates (OCR), we could evaluate two key parameters: Spare Respiratory Capacity (SRC), which indicates flexibility and reserve capacity to respond to sudden increases in energy demands and Maximal Respiration Capacity (MRC), which represents the highest capacity of mitochondrial respiration under full stimulation. Exposure of CD4^+^ T cells to increasing concentration of Zaprinast (4, 16, 100 µM) led to an immediate, dose-dependent inhibition of mitochondrial respiration as reflected by reduction in both MRC (up to 36% reduction) and SRC (up to 77% reduction), with a stronger effect observed at 100µM (Fig. 3a). Notably, the lowest concentration tested, 4 µM, corresponding to the dose used in our latency-reversal experiments, already produced a significant decrease in OCR, with a 23.97% reduction in MRC and a 54.32% reduction in SRC, while higher concentrations did not yield proportionally greater MCP inhibition. We therefore selected 4 µM Zaprinast as the benchmark dose for subsequent metabolic studies, thereby minimising the risk of off-target effects.

We then compared the immediate impact of Zaprinast to longer exposure (18 h). After incubating CD4^+^ T cells with DMSO (control) or Zaprinast for 18 h, we tested three conditions in parallel: immediate Zaprinast impact, 18 h of Zaprinast treatment and its reversibility (Zaprinast removal after 18 h of incubation and replacement with DMSO) (Fig. 3b). The immediate and direct effect of 4 µM Zaprinast reduced mitochondrial respiration, as observed above (Fig. 3c). In contrast, 18 h of exposure resulted in a moderate and significant increase in basal respiration (16%), with a concomitant increase in MRC (28%) and SRC (28%), although the latter was not statistically significant due to donor-to-donor sample variations. This increase outlines that the mitochondrial respiration was restored and functional after 18 h of Zaprinast treatment. It could be the result of T-cell metabolic plasticity, where cells compensate for reduced mitochondrial Pyruvate entry by using alternative carbon sources like glutamate or fatty acid (FA) to maintain TCA cycle function, as observed in previous studies [37, 40, 43, 45, 46]. Remarkably, Zaprinast removal after 18 h led to a significant 41% increase in basal respiration with even greater upregulations of MRC (83%) and SRC (89%), though the latter two were not significant due to sample-to-sample variations (Fig. 3c). As Zaprinast is a non-covalent inhibitor, the observed boost in mitochondrial respiration upon its removal could be attributed to Pyruvate re-entry into the mitochondria, combined with other nutriments, all converging to fuel the TCA cycle.

### Zaprinast treatment promotes a shift towards aerobic glycolysis in primary CD4^+^ T cells

Under basal conditions, pyruvate, the end product of glycolysis, primarily serves as a source of acetyl-CoA in the mitochondria for the TCA cycle and OXPHOS. Inhibiting pyruvate entry into mitochondria with Zaprinast is expected to lead to its cytoplasmic accumulation. We examined whether Zaprinast treatment was indeed promoting a shift toward aerobic glycolysis by redirecting cytoplasmic pyruvate into lactate formation and secretion. As previously described, we examined Zaprinast effects on CD4^+^ T cells from blood donors under 3 conditions tested in parallel: immediate effect, 18-hour treatment and its reversibility after 18-hour treatment (Fig. 3b). To assess lactate secretion, we measured extracellular acidification rate (ECAR) (Fig. 3d). Zaprinast induced a rapid and significant 1.55-fold increase in ECAR, indicating enhanced lactate production. This upregulation was sustained after 18 h of Zaprinast treatment, showing a 1.41-fold increase compared to control (DMSO) (Fig. 3d). Interestingly, ECAR returned to basal levels upon drug removal after 18 h, demonstrating the reversible action of Zaprinast on CD4^+^ T-cell metabolism.

To further assess the impact of Zaprinast on aerobic glycolysis in CD4^+^ T cells, we monitored glucose uptake via intracellular accumulation of 2-NBDG (a fluorescent glucose analogue) by flow cytometry after 18 h of treatment. Zaprinast induced a significant 2.04-fold increase in glucose uptake compared to DMSO (*p* = 0.0030) (Fig. 3e). By comparison, global T-cell activation mediated by PMA/Ionomycin treatment (positive control) induced a similar 2.3-fold upregulation of 2-NBDG staining after 18 h (*p* = 0.0333) (Fig. 3e). This enhanced glucose uptake correlates with the increased aerobic glycolytic activity observed above.

### Zaprinast boosts energy profile of CD4^+^ T cells without inducing ROS production

We investigated the effects of Zaprinast treatment on the capacity of resting CD4^+^ T cells from blood donors to mobilise their resource and metabolism in response to stress using the Seahorse XF ATP-real time assay. This assay presents the advantage of simultaneously evaluating the contribution of glycolysis and mitochondrial respiration to ATP production in response to stress. ATP production was measured immediately or after 18 h of Zaprinast treatment. Immediate impact of Zaprinast treatment resulted in a 0.8-fold reduction in mitochondrial ATP (MitoATP) production (*p* = 0.0278) compared to DMSO, while Glycolytic ATP (GlycoATP) production remained stable. After 18 h of Zaprinast treatment, MitoATP significantly increased by 1.41-fold compared to DMSO control (*p* = 0.0273) (Fig. 3f). Similarly, GlycoATP production showed a significant 1.32-fold increase compared to DMSO (*p* = 0.0288) (Fig. 3f). These findings further demonstrate that Zaprinast induces a time-dependent reprogramming of resting CD4^+^ T cells, ultimately enhancing their ATP production capacity in stress conditions through both glycolytic and mitochondrial pathways.

Given the impact of Zaprinast on mitochondrial function, we monitored whether Zaprinast could alter the mitochondrial membrane potential or ROS production (Supplementary Fig. 2a and 2b). While we observed a moderate but not significant reduction of mitochondrial membrane potential following Zaprinast treatment over time, it was not associated with an increase in ROS production.

Metabolic profiling of Zaprinast-treated CD4^+^ T cells revealed a biphasic response where an initial rapid decline in mitochondrial respiration is followed by a sustained phase characterised by concurrent upregulation of OXPHOS and anaerobic glycolysis, similar to a Warburg-like metabolic reprogramming.

## Discussion

**Fig. 4:**
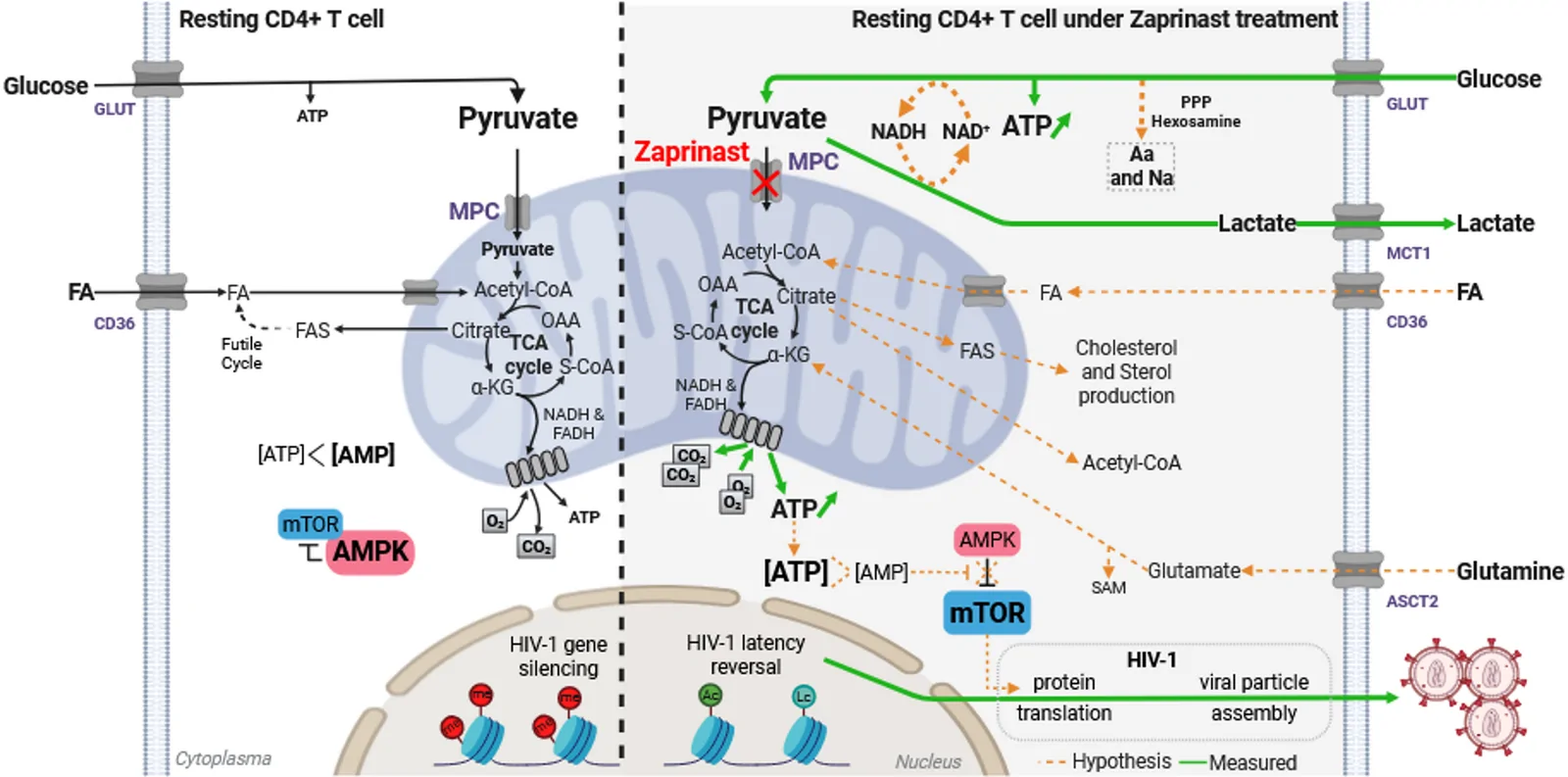
Zaprinast-induced metabolic reprogramming of resting CD4^+^ T cells promote HIV-1 latency reversal. Graphical representation of the metabolic changes in resting CD4^+^ T cells without treatment (left) and after Zaprinast treatment (right). In the basal state, resting CD4^+^ T cells primarily rely on oxidative phosphorylation (OXPHOS) for ATP production, with naïve cells utilising glycolysis to fuel the tricarboxylic acid (TCA) cycle and memory cells employing both glycolysis and fatty acid oxidation. Zaprinast treatment blocks pyruvate entry in the mitochondria and induces a metabolic shift, redirecting pyruvate towards lactate formation and secretion. After 18 h of treatment, there is an increase in overall metabolic activity, including enhanced glycolysis, elevated mitochondrial respiration, and increased ATP production, supported by increased glucose uptake. Potential metabolic compensation through fatty acid oxidation or glutaminolysis may occur to sustain the TCA cycle and mitochondrial respiration. This metabolic reprogramming ultimately leads to HIV-1 latency reversal evidenced by the production of HIV-1 RNA and viral particles. Green arrows indicate the observed effects of Zaprinast, while dashed orange arrows represent potential effects based on literature review. *FA* (Fatty acid), *FAS* Fatty Acid Synthesis, *TCA* Tricarboxylic Acid Cycle, *OAA* Oxaloacetate, *S-CoA* Succinyl-CoA, *α-KG* α-Ketoglutaric acid, *Aa* Amino Acid, *Na* Nucleic Acid, *me* methyl group, *Ac* acetyl group, *Lc* Lactyl group, *SAM* S-Adenosyl methionine, *PPP* Pentose Phosphate Pathway

This study identifies Zaprinast, an MPC inhibitor, as a novel and interesting addition to the repertoire of LRAs capable of reactivating HIV-1 from latent reservoirs in ART-suppressed individuals ex vivo. Using bona fide HIV-1 reactivation assays, we showed that Zaprinast induced HIV-1 gene expression leading to viral particle production in circulating reservoirs from ART-treated and virally suppressed individuals, ex vivo. Across multiple primary cell-based models and donors, Zaprinast elicited moderate but consistent increases in both cell-associated US and MS RNA and virion release, with response patterns that differed from those observed with strong TCR stimulation and the HDAC inhibitor Romidepsin or SAHA, underscoring the multifactorial nature of HIV latency and the presence of distinct mechanistic blocks across individuals and reservoirs [61]. Metabolic analyses revealed that Zaprinast triggered a biphasic metabolic reprogramming, initially restricting pyruvate entry into mitochondria to transiently suppress OXPHOS, while simultaneously promoting aerobic glycolysis by redirecting the cytosolic pyruvate towards lactate production, followed by restoration of mitochondrial respiration, likely via anaplerotic replenishment of the TCA cycle through alternative substrates such as glutamine-derived α-ketoglutarate or fatty acid β-oxidation as previously described for Zaprinast and other MPCis (Fig. 4) [45, 46, 62].

Taken together, our data support a model in which Zaprinast mediated latency reversal via a metabolic rewiring of CD4 T cells, which occurred in the absence of T cell activation, proliferation, or redox imbalance. Beyond this primary metabolic shift, the downstream mechanisms linking Zaprinast rewiring to HIV-1 reactivation remain incompletely defined. Pathways branching from glycolysis, including the TCA cycle, the pentose phosphate pathway, or gluconeogenesis, could provide intermediate metabolites and cofactors, such as acetyl-CoA, α-ketoglutarate, succinate, NAD⁺, and ATP, that modulate chromatin-modifying enzymes, transcriptional regulation, and post-transcriptional processes such as splicing and nuclear export, all of which have been implicated in the maintenance and reversal of HIV-1 gene silencing in viral reservoirs [27, 61, 63–66]. Although our study was designed primarily to establish proof-of-concept for metabolism-driven latency modulation, an important avenue for future work will be to compare Zaprinast with canonical epigenetic LRAs or PKC agonists at the level of histone marks and chromatin remodelers, and to determine whether Zaprinast also influences splicing, nuclear export or translation of viral transcripts.

Notably, Zaprinast effects were reversible, with CD4^+^ T cells resuming an OXPHOS-driven quiescent metabolic state upon drug removal and were not associated with cytotoxicity, oxidative stress, or non-specific T-cell activation or proliferation, thereby fulfilling key requirements for candidate LRAs and supporting strategies targeting the quiescent metabolism of CD4^+^ T cells as a promising component of “Shock and Kill” strategies.

Emerging evidence highlights the dynamic interplay between glycolysis and OXPHOS as a metabolic checkpoint governing the establishment and maintenance of HIV-1 latency and the persistence of viral reservoirs [29]. If low basal glycolytic activity limits HIV-1 reactivation, HIV-1 latency reversal must also be supported by functional mitochondria [12, 14, 15, 26–28]. Zaprinast biphasic metabolic reprogramming meets dual requirements for latency reversal by promoting glycolytic bursts while maintaining mitochondrial respiration to meet energetic and biosynthetic needs for viral biosynthesis. These findings underscore that latency reversibility depends on coordinated glycolytic-OXPHOS synergy, offering a framework for designing metabolically targeted HIV-1 cure interventions.

This study suggests pyruvate metabolism as a potential checkpoint for HIV-1 latency reversal, with the MPC as a potential therapeutic target. In resting T cells, pyruvate, end product of glucose oxidation, is transported across the inner mitochondrial membrane via the MPC, a heterodimeric transmembrane complex (MPC1/MPC2), to generate acetyl-CoA for the TCA cycle (Fig. 4) [67, 68]. Alternatively, at this branching point, cytosolic pyruvate can be converted to lactate by lactate dehydrogenase (LDH), regenerating NAD^+^ in the process to sustain and promote glycolysis (Fig. 4) [69]. Because the pool of cytosolic pyruvate is a key junction where glycolysis, OXPHOS, and mitochondrial dynamics intersect, MPC has been identified as a potential therapeutic target in metabolic disorders, including type 2 diabetes, obesity, and cancers and MPC inhibitors as potential therapeutic agents [70–72].

Zaprinast was originally developed as a cGMP-specific phosphodiesterase (PDE) inhibitor with reported IC50 values in the micromolar range (200 µM) for PDE5/6 and other PDE isoforms. Subsequently, work by Du et al. demonstrated that Zaprinast is in fact a potent inhibitor of the MPC, with an IC50 of approximately 321nM, indicating that MPC is a primary pharmalogical target and that PDE inhibition can represent an off-target in some settings [38]. Later, Elhammaji et al. identified inhibition of glutaminase and consequently modulation of the 2HG metabolic pathway as an additional off-target activity, requiring concentration around 100 μm to achieve substantial effects [41, 42, 44, 73]. In our study, we selected 4 µM Zaprinast, roughly one order of magnitude above the MPC IC50 to facilitate efficient cellular uptake and near-maximal MPC inhibition, yet below the IC50 values reported for PCEs and the 2HG/GLS pathways, thereby providing robust MPC engagement in resting CD4^+^ T cells while minimising the likelihood of confounding off-target activities. Its capacity to block pyruvate entry into the mitochondria and to reprogramme cellular metabolism has been demonstrated in a variety of cell types, including retina, brain tissue, primary hepatocytes, and neuroblastoma cells [38, 44, 73, 74]. Other specific MPC inhibitors, such as UK-5099, thiazolidinediones (pioglitazone and rosiglitazone), and, more recently, 7ACC2 and Mito-66, have also been used as metabolic modulators [75–77]. While off-target effects cannot be excluded [73, 78, 79], the observed immediate inhibition of mitochondrial respiration and increase in lactate secretion are consistent with the inhibitory effects of Zaprinast on the MPC. Zaprinast was selected over covalent MPCi like UK-5099, due to its nanomolar potency, reversible binding to MPC, and proven capacity in improving glucose tolerance in preclinical models, a key advantage for clinical translation [44, 80]. More extensive dose–response studies on viral endpoints will be required in future dedicated pharmacology work to define the full pharmacodynamic profile of Zaprinast latency reversal activity.

While this study focuses on circulating CD4^+^ T cells, future work should assess Zaprinast impact on tissue reservoirs (gut-associated lymphoid tissue, adipose tissue, CNS) associated with distinct metabolic niches, and non-T cell populations, including macrophages and microglial cells as those present distinct metabolic environments [81–85]. Additionally, the effects of Zaprinast on CD8^+^ T cell antiviral functions, which are important for viral clearance, warrant evaluation as CD8^+^ T cell functionality is tightly associated with mitochondrial metabolism [85–87]. Previous studies have suggested that metabolic modulation, through MPC inhibition, can enhance immune functions, but whether Zaprinast impairs or improves CD8^+^ T cell activity in the context of HIV-1 cure remains to be evaluated [46].

By demonstrating that pharmacological MPC inhibition can reverse HIV-1 latency in preclinical models, the present study establishes a foundational framework for reducing viral reservoirs through immunometabolic fine-tuning. Future work should explore combinatorial strategies pairing MPC inhibition with other classes of LRAs or immunotherapies to maximise HIV-1 reservoir reduction.

## Materials and methods

### Participants recruitment and blood collection – Table 1

This study included 38 PLWH on suppressive ART with undetectable viremia (Table 1). Participants of the HIVCURE Cohort were recruited at Mater Misericordiae University Hospital (MMUH) and Saint Vincent’s University Hospital (SVUH) in Dublin, Republic of Ireland. All participants were adults and provided written informed consent. This study received ethical approval (RS19-004, 1/378/1808). Blood was collected in 9 mL EDTA-tubes (Sarstedt #02.1066.001). All experiments related to HIV-1 were performed according to BSL3 guidelines and performed in a CL3 laboratory.

De-identified buffy coats were provided by the Irish Blood Transfusion Service (IBTS) for research use. All samples were obtained with donor consent for use in research and were approved by the UCD Human Research Ethics Committees (LS-LR-22-246-dAutume-Gautier).

### Primary cell isolation

CD4^+^ T cells were isolated using the EasySep™ Direct Human CD4^+^ T Cell Isolation Kit (Stemcell Technologies, #19662). CD8-depleted PBMC were isolated using the Rosette Sep CD8^+^ T cell depletion kit (Stemcell Technologies, #15663). CD4^+^ T cell purity was tested following isolation using PerCP CD3 antibody (Biolegend, #300428) and CD4 antibody, anti-human FITC (Miltenyi Biotec, #130-113-213).

### In vitro HIV-1 reactivation assay - cell culture and drug treatments

 In vitro HIV-1 latency assays were performed with primary CD4^+^ T cells according to the Planelles model with some modifications [47]. Briefly, isolated primary CD4^+^ T cells were activated with soluble anti-CD3/anti-CD28 antibodies (25 µL/mL (Stemcell Technologies, #10991)) and non-polarising treatment (10 ng/mL human TGFb1 (Miltenyi Biotec, #130-095-067), 1 µg/mL anti-IL4 antibody (Miltenyi Biotec, #130-095-753), 2 µg/mL anti-IL12 antibody (Miltenyi Biotec, #130-095-755)). Following stimulation and non-polarising treatment, CD4^+^ T cells were infected in vitro with HIV-1 (89.6) and maintained in culture over several weeks with IL-2 and ART. Cells were cultured in RPMI 1640 (Gibco, #61870010) + 10% FBS (Gibco, #10500-064) + 1% Pen-strep (Gibco, #15140122) at 1*10^6^ cells/mL with 10 U/mL IL-2 (Miltenyi Biotec, #130-09-745). On day 6, cells were spinoculated with HIV-1 (89.6) and maintained in culture with 10 µM Saquinavir (Sigma-Aldrich, #S8451-50 mg) and 10 µM Efavirenz (SelleckChem, #S4685) and IL-2 (Miltenyi Biotec, #130-09-745) to establish in vitro HIV-1 latency. On days 21 and 28, the memory phenotype was evaluated as described in a later section. On day 28, HIV-1 reactivation assays were conducted in a 96-well plate where cells were treated with DMSO (Sigma-Aldrich, #D4540),100 µl/mL anti-CD3/anti-CD28 antibodies, with 40 U/mL IL-2 (Miltenyi Biotec, #130-09-745) or 4 µM Zaprinast (Sigma-Aldrich, #684500-25MG) for 24 h.

### In vitro HIV-1 reactivation assay - RNA extraction and HIV-1 total RNA quantification

After 24 h of treatment, cells and supernatants were directly lysed by osmotic shock with DEPC water at 56 °C for 30 min. A semi-nested RT-qPCR was performed using previously described primers and probes targeting the polyA tails of viral transcripts (Supplementary Table 1) [88]. Briefly, a first-round RT-PCR was performed using the Verso RT-PCR kit on Thermocycler 2027 (AppliedBiosystem, #272S7121870). Cycling conditions were as follows: *30 min 50 °C*,* 15 min 95 °C*,* 10X [30 Sec 95 °C*,* 1 min 54 °C*,* 1 min 72 °C]*,* 10 min 72 °C*. A second round qPCR was performed using Quantinova probe qPCR (Qiagen, #208252) on Quantstudio 5 (AppliedBiosystem, #A28134). Cycling conditions were as follows: *30 min 50 °C*,* 10 min 95 °C*,* 50X [15 Sec 94 °C*,* 1 min 60 °C]*. Quantification of the qPCR over DMSO was calculated using the Pfaffl calculation methods [89].

### In vitro primary model of HIV-1 using VSV-G pseudotyped particles

PBMCs were isolated from buffy coats of 6 healthy HIV seronegative donors, obtained from the “Centre de transfusion de Charleroi”, by Ficoll density gradient centrifugation (Stem Cell Technologies, #18061). CD4^+^ T lymphocytes were negatively selected (Invitrogen, #8804 − 6811), according to the manufacturer’s instructions. Isolated CD4^+^ T cells were then infected by spinoculation (2 h, 800× g, 32 °C) with VSV-G pseudotyped HIV-1 strain NL4.3 viral particles at a multiplicity of infection (MOI) of 1000. Following infection, cells were allowed to recover for 2 h, washed two times, and cultured in RPMI 1640 (Gibco, #61870010) + 10% FBS (Gibco, #10500-064) + 1% Pen-strep (Gibco, #15140122) at 2 × 10^6^ cells/ml supplemented with 30 U/ml IL-2 (Proteintech, #HZ-1015) and 5µM of the protease inhibitor saquinavir (MedChemExpress, #HY-17007) to establish in vitro HIV-1 latency. At day 3 post-infection, HIV-1 reactivation assays were conducted in a 6-well plate where cells were treated with DMSO (Sigma-Aldrich, #D4540) or 4 µM Zaprinast (Sigma-Aldrich, #684500-25MG) for 72 h in presence of 30 U/mL IL-2 and 5µM saquinavir. As control, CD4 + T cells were treated with 5nM romidepsin (MedChemExpress, #HY-15149) for 24 h.

### In vitro HIV-1 expression induction – RNA extraction and HIV-1 US and MS RNA quantification

Total RNA samples were isolated using the Quick-DNA/RNA Miniprep Plus Kit (Zymo, #D7003) according to the manufacturer’s protocol. Reverse transcription was performed with HiScript III RT SuperMix for qPCR following the manufacturer recommendation (Vazyme, #R323-01). cDNAs were quantified by quantitative PCR using Luna Universal qPCR Master Mix (NEB, #M3003S) in the QuantStudio 3 Real-Time PCR System (Applied Biosystems). Both MS and US HIV-1 mRNA quantity were quantified and normalized relative to the amount of YWHAZ, and TBP cellular mRNA. The sequences of the primers used for quantification are listed in Supplementary Table 1.

### Ex vivo HIV-1 reactivation assay with CD4^+^ T cells isolated from PLWH on suppressive ART - cell culture and drug treatments

Isolated CD4^+^ T cells were cultured in RPMI 1640 Medium, GlutaMAX™ Supplement (Gibco, #61870010) supplemented with 10% FBS (Gibco, #10500-064) and 1% Pen-strep (Gibco, #15140122) at 2*10^6^ cells/mL in the presence of 100 nM Nevirapine (Sigma-Aldrich, #SML0097) and 16 µM Saquinavir (Sigma-Aldrich, #S8451-50 mg). Cells were stimulated for 3 days with DMSO (Sigma-Aldrich, #D4540), 4 µM Zaprinast (Sigma-Aldrich, #684500-25MG) or 20 nM PMA (Merck, #P8139-1MG) and 280 nM Ionomycin (Sigma-Aldrich, #I0634).

### Ex vivo HIV-1 reactivation assay with CD4^+^ T cells isolated from PLWH on suppressive ART - RNA extraction and quantification

CA RNAs were extracted using the RNeasy Mini Kit (Qiagen, #74106) followed by a DNase I digestion step (Turbo DNA-free kit, Invitrogen #AM1907). A semi-nested Reverse Transcription - quantitative PCR was performed to measure multi-spliced *tat-rev* transcripts as previously described (Supplementary Table 1) [90]. The first-round reverse transcription was performed using the Verso RT-PCR kit (Sigma-Aldrich, #I0634) on Thermocycler 2027 (AppliedBiosystem, #272S7121870). Cycling conditions were as follows: *15 min 50 °C; 2 min 95 °C; 16X [20 Sect. 95 °C; 30 Sect. 60 °C; 1 min 72 °C]; 5 min 72 °C.* A second round qPCR was performed in duplicate using the Quantinova probe qPCR kit (Qiagen, #208252) on Quantstudio 5 (AppliedBiosystem, #A28134). Cycling conditions were as follows: *15 min 50 °C; 2 min 95 °C; 16X [20 Sect. 95 °C; 30 Sect. 60 °C; 1 min 72 °C]; 5 min 72 °C*. RT-qPCR was performed targeting the housekeeping gene *TATA box-binding protein* (TBP) using the QuantiTect Probe RT-PCR kit (Qiagen, #204445) on Quantstudio 5 (AppliedBiosystem, #A28134) with the primers and probes previously published (Supplementary Table 1) [91]. Cycling conditions were as follows: *2 min 50 °C; 15 min 95 °C*,* 45X [45 Sect. 95 °C; 1 min 60 °C].* Three of the eleven samples tested showed spontaneous reactivation in DMSO controls and were excluded from the analysis to avoid confounding by Zaprinast mediated-signal amplification of residual and pre-existing HIV-1 transcriptional activity. A non-template control well was always included and negative. All qPCRs were analysed using Design&Analysis Software (Thermofisher Scientific V2.6.0). For each PCR plate, the mean PCR efficiency was calculated using LinRegPCR (V2021.2) [92]. Then, the relative expression of *tat-rev* HIV-1 MS RNA was calculated for each treatment compared to DMSO using the following Pfaffl methods [89].

### Ex vivo HIV-1 reactivation assay with CD8-depleted PBMC isolated from PLWH on suppressive ART - Cell culture and drug treatments

CD8-depleted PBMCs were resuspended at 2*10^6^ cells/mL in RPMI 1640 Medium, GlutaMAX™ Supplement (Gibco, #61870010) supplemented with 10% FBS (Gibco, #10500-064) and 1% Pen-strep (Gibco, #15140122). Cells were stimulated for up to 8 days with DMSO (Sigma-Aldrich, #D4540), 4 µM Zaprinast (Sigma-Aldrich, #684500-25MG), 25 µL/mL anti-CD3/anti-CD28 antibodies (Stemcell Technologies, #10971) and 10 U/mL IL-2 (Miltenyi Biotec, #130-09-745).

### Ex vivo HIV-1 reactivation assay with CD8-depleted PBMC isolated from PLWH on suppressive ART - RNA extraction and viral loads quantification

Viral gRNA was extracted from cell-free supernatants, collected on day 5 and day 8, using the QIAamp Viral RNA Mini Kit (Qiagen, #52904) and stored at -80 °C. A one-step RT-qPCR was performed with the previously published primers and probes targeting the HIV-1 gag region (Supplementary Table 1) [59], using Quantitect probe RT-qPCR (Qiagen, #204443) on Quantstudio 5 (AppliedBiosystem, #A28134). Cycling conditions were as follows: 30 min 50 °C, 10 min 95 °C, 50X [15 Sect. 94 °C, 1 min 60 °C]. Absolute quantification was performed with a standard curve of viral particles produced by ACH2 cell line stimulated for 48 h with 10 ng/mL TNFα (Sigma-Aldrich, SRP3177-50UG) [93]. Of the 24 samples, 7 that displayed spontaneous HIV-1 reactivation in DMSO controls were excluded. In addition, when HIV reactivation was detected under Zaprinast treatment at day 5, analysis was not extended to day 8.

An HIV-1 p24 ELISA assay was performed to quantify the viral particle concentration of the virus stock following the instructions of the manufacturer (Aalto Bio Reagents, Supplementary Table 1). The viral stock was measured with a concentration of 1 ng/mL of p24, corresponding to 10^7^ viral particles per mL. A non-template control well was always included and negative. All qPCRs were analysed using Design&Analysis Software (Thermofisher Scientific, V2.6.0).

### Flux analysis – Seahorse analyser

OCR and ECAR of CD4^+^ T cells were measured using the Seahorse XF Pro Analyzer (Seahorse Biosciences, Agilent) or Seahorse HF HS Mini Analyzer (Seahorse Biosciences, Agilent) as previously described [94]. Cells were plated in Seahorse XFe96/XF Pro FluxPack (Agilent, #103792-100) or Seahorse XF Pro M FluxPak Mini (Agilent, #103723-100). Each experiment was analysed using the Agilent application Seahorse Analytics (Agilent, V1.0.0-699).

### Mitostress test

Following CD4^+^ T cells isolation from blood donors, cells were stimulated for 18 h with DMSO (Sigma-Aldrich, #D4540) or 4 µM Zaprinast (Sigma-Aldrich, #684500-25MG) in RPMI 1640 (Gibco, #11875101) supplemented with 10% FBS (Gibco, #10500-064) and 1% Pen-strep (Gibco, #15140122). 2*10^5^ CD4^+^ T cells were seeded per well in Seahorse XF RPMI assay medium pack, pH 7.4 (Agilent #103680-100) supplemented with 10 mM Seahorse XF Glucose (Agilent, #103577-100), and 2 mM Seahorse XH L-Glutamine (Agilent, #103579-100). Then, an hour incubation at 37 °C without CO_2_ was performed. The Mitostress test assay was performed in triplicate with the sequential injection of compounds as follows in the different ports: (A) DMSO (Sigma-Aldrich, #D4540) or 4 µM Zaprinast (Sigma-Aldrich, #684500-25MG); (B) 2.5 µM Oligomycin (Agilent); (C) 2 µM FCCP (Agilent); (D) 0.5 µM Rotenone/Antimycin (Agilent).

### ATP-real time

Following CD4^+^ T cells isolation from blood donors, cells were stimulated for 18 h with DMSO (Sigma-Aldrich, #D4540) or Zaprinast 4µM (Sigma-Aldrich, #684500-25MG) in RPMI 1640 (Gibco, #11875101) supplemented with 10% FBS (Gibco, #10500-064) and 1% Pen-strep (Gibco, #15140122). 2*10^5^ CD4^+^ T cells were washed and seeded per well in Seahorse XF RPMI assay medium pack, pH 7.4 (Agilent, #103680-100) supplemented with 10 mM Seahorse XF Glucose (Agilent, #103577-100), and 2 mM Seahorse XH L-Glutamine (Agilent, #103579-100). Then, an hour incubation at 37 °C without CO_2_ was performed. The ATP-Real Time assay was performed in triplicate with the sequential injection of compounds as follows in the different ports: (A) DMSO (Sigma-Aldrich, #D4540) or 4 µM Zaprinast (Sigma-Aldrich, #684500-25MG); (B) 2.5 µM Oligomycin (Agilent); (C) 0.5 µM Rotenone/Antimycin (Agilent).

### Extracellular acidification measurement – pH Xtra

2*10^6^ CD4^+^ T cells from blood donors were plated per well in a 96-well plate format and stimulated for 18 h with DMSO (Sigma-Aldrich, #D4540), 4 µM Zaprinast (Sigma-Aldrich, #684500-25MG). Cells were washed and resuspended in pH Buffer (pH Xtra, Agilent #Ph200-4). Extracellular acidification was measured in real-time over 2 h using pH-Xtra Glycolysis Assay (Agilent, #PH-200-4) on a Multi-Mode Microplate Readers (Molecular Device ID3, 380 nm / 615 nm). DMSO and Zaprinast were refreshed during the wash, and Antimycin A (Sigma-Aldrich, #A8674) or 2-Deoxy-D-glucose (Sigma-Aldrich, #D6134) were added after. Each condition was performed in duplicate. The data was analysed using the MitoXpress Xtra & pH-Xtra Data Visualization Tool provided by Agilent [95]. From the Extracellular acidification measurement, a slope was calculated and normalised over DMSO [96–99].

### Flow cytometry platform and analysis

Flow cytometry was performed on the Cytoflex S V4-B2-Y4-R3 Flow Cytometer (Beckman Coulter, #B75408) and the Cytoflex V2-B4-R2 Flow Cytometer (Beckman Coulter, #C02945). The data were analysed on Cytexpert software (Beckman Coulter, V2.4.0.28). Mean Fluorescence Intensity (MFI) was measured in each experiment using Cytoflex S. Cells were washed in DPBS, no calcium, no magnesium (Gibco, #14190094). Following manufacturer instructions, cells were fixed with BD Cytofix/Cytoperm fixation/permeabilisation Kit (BD Biosciences, #554714) and resuspended in DPBS, no calcium, no magnesium (Gibco, #14190094) and 1% EDTA (Sigma-Aldrich, #03690-100mL). Mean Fluorescence Intensity (MFI) was measured using Cytoflex S, and the percentage of stained cells compared to DMSO treatment was measured.

### Glucose uptake − 2-NBDG staining

Following CD4^+^ T cell isolation from blood donors, 7,25*10^5^ CD4^+^ T cells per well were stimulated for 18 h at 2.10^6^ CD4^+^ T cell/mL with DMSO (Sigma-Aldrich, #D4540), 20 nM PMA (Merck, #P8139-1MG) and 280 nM Ionomycin (Sigma-Aldrich, #I0634), or 4 µM Zaprinast (Sigma-Aldrich, #684500-25MG) in RPMI 1640 (Gibco, #11875101) supplemented with 10% FBS (Gibco, #10500-064) and 1% Pen-strep (Gibco, #15140122). Then, cells were incubated for 1 h with 50 µM of 2-NBDG (Invitrogen, #N13195) in RPMI1640 medium; no glucose (Gibco, #11879020).

### Mitochondrial staining

Following CD4^+^ T cell isolation from blood donors, cells were stimulated for 72 h at 2*10^6^ cells/mL with DMSO (Sigma-Aldrich, #D4540), 20 nM PMA (Merck, #P8139-1MG) and 280 nM Ionomycin (Sigma-Aldrich, #I0634), or 4 µM Zaprinast (Sigma-Aldrich, #684500-25MG) in RPMI 1640 (Gibco, #11875101) supplemented with 10% FBS (Gibco, #10500-064) and 1% Pen-strep (Gibco, #15140122). A 30-minute incubation was performed for the immediate treatment and 50 µM FCCP treatment (negative control for membrane potential). Cells were collected on day 1, day 3 and day 6 in triplicate in a 96-well plate (Corning, #353077). Cells were stained with 100 nM MitoTracker™ Red CMXRos (Invitrogen, #M7512) for 30 min. Then, cells were fixed as previously described and stained with 200 nM Mitoview Green (Generon, #70054) for 30 min. Cells were then washed and resuspended as previously described. MFI was measured using Cytoflex S, and FCCP MFI were subtracted from the corresponding treatment to exclude the background from the signal. Ratio potential/mass was calculated on each day/treatment and then normalised over the corresponding DMSO ratio:

$$\:Ratiopotentia/mass=\frac{{MFI}_{membrane\:potentia}}{{MFI}_{mitochondrial\:mass}}$$

### Reactive oxygen species staining

Following CD4^+^ T cell isolation from blood donors, cells were stimulated for 72 h at 2*10^6^ cells/mL with DMSO (Sigma-Aldrich, #D4540), or 4 µM Zaprinast (Sigma-Aldrich, #684500-25MG) in RPMI 1640 (Gibco, #11875101) supplemented with 10% FBS (Gibco, #10500-064) and 1% Pen-strep (Gibco, #15140122). A 30-minute incubation was performed for the immediate treatment and 100 µM Antimycin (Sigma-Aldrich, #A8674). Cells were collected on day 1, day 3 and day 6 in a 96-well plate (Corning, #353077). Cells were stained with 5 µM MitoSOX™ Red Mitochondrial Superoxide Indicators (Thermofisher, #M36007). Then, cells were fixed and resuspended as previously described.

### CD4^+^ T cell viability staining

Following CD4^+^ T cell isolation from blood donors, cells were stimulated up to 6 days at 2*10^6^ cells/mL with DMSO (Sigma-Aldrich, #D4540), 20 nM PMA (Merck, #P8139-1MG) and 280 nM Ionomycin (Sigma-Aldrich, #I0634), 4 µM Zaprinast (Sigma-Aldrich, #684500-25MG in RPMI 1640 (Gibco, #11875101) supplemented with 10% FBS (Gibco, #10500-064) and 1% Pen-strep (Gibco, #15140122). Cells were collected on day 1, day 3 and day 6 in a 96-well plate (Corning, #353077). Cells were stained with Viobility 405 nm/452 nm Fixable Dye in DPBS, no calcium, no magnesium (Gibco, #14190094) following the manufacturer instructions. Then, cells were washed in DPBS, no calcium, no magnesium (Gibco, #14190094) and fixed with BD Cytofix/Cytoperm fixation/permeabilisation Kit (BD Biosciences, #554714). Then, cells were resuspended in DPBS, no calcium, no magnesium (Gibco, #14190094) and 1% EDTA (Sigma-Aldrich, #03690-100mL).

### CD4^+^ T cell proliferation staining

Following CD4^+^ T cell isolation from blood donors, cells were stained for 5 min with 5 µM CellTrace™ CFSE Cell Proliferation Kit (Invitrogen, #C34554), then washed and stimulated up to 6 days at 2*10^6^ cells/mL with DMSO (Sigma-Aldrich, #D4540), 25 µL/mL ImmunoCult™ Human anti-CD3/anti-CD28 antibodies (Stemcell Technologies, #10991), 4 µM Zaprinast (Sigma-Aldrich, #684500-25MG) in RPMI 1640 (Gibco #11875101) supplemented with 10% FBS (Gibco, #10500-064) and 1% Pen-strep (Gibco, #15140122). Cells were collected after 6 days in a 96-well plate (Corning, #353077). Then, cells were fixed with BD Cytofix/Cytoperm fixation/permeabilisation Kit (BD Biosciences, #554714) and resuspended in DPBS, no calcium, no magnesium (Gibco, #14190094) and 1% EDTA (Sigma-Aldrich, #03690-100mL). MFI was measured using Cytoflex S, and the percentage of proliferating cells was calculated as indicated by the manufacturer.

### Surface staining – CD4^+^ T cell staining

Following CD4^+^ T cell isolation from blood donors, cells were stained with PerCP anti-human CD3 (Biolegend, #300428) and CD4 (Miltenyi Biotec, #130-113-213) in DPBS, no calcium, no magnesium (Gibco, #14190094) for 30 min.

Following CD4^+^ T cell isolation, cells were stimulated up to 6 days at 2*10^6^ cells/mL with DMSO (Sigma-Aldrich, #D4540), 20 nM PMA (Merck, #P8139-1MG) and 280 nM Ionomycin (Sigma-Aldrich, #I0634), 4 µM Zaprinast (Sigma-Aldrich, #684500-25MG in RPMI 1640 (Gibco #11875101) supplemented with 10% FBS (Gibco, #10500-064) and 1% Pen-strep (Gibco, #15140122). Cells were collected on day 1, day 3 and day 6 in a 96-well plate (Corning #353077) and stained with APC/Cyanine7 anti-human CD25 Antibody (Biolegend, #302614) and PE anti-human CD69 Antibody (Biolegend, #310906) in DPBS, no calcium, no magnesium (Gibco, #14190094) for 30 min. Then, cells were fixed and resuspended as previously described.

### Surface staining – CD8-depleted PBMC memory phenotype staining

Following CD8-depleted PBMC, from blood donors, stimulation with anti-CD3/anti-CD28 antibodies and spinoculation, the memory phenotype was evaluated on day 21. 1*10^6^ cells were collected in a 96-well plate (Corning #353077). Cells were stained for 10 min at 4 °C with 5 µL of each following antibody (CD25-APC (Miltenyi Biotec, #130-101-435), CD69-PE (Miltenyi Biotec, #130-099-382), CD197-Vio770 (Miltenyi Biotec, #130-099-164), CD27-Vioblue (Miltenyi Biotec, #130-099-502), CD45RA-PerCP (Miltenyi Biotec, #130-095-466), CD45R0-FITC (Miltenyi Biotec, #130-095-462)).

Following CD8-depleted PBMC stimulation with anti-CD3/anti-CD28 antibodies and spinoculation, the cells were treated for up to 8 days with DMSO (Sigma-Aldrich, #D4540), 4 µM Zaprinast (Sigma-Aldrich, #684500-25MG), 25 µL/mL anti-CD3/anti-CD28 antibodies (Stemcell Technologies, #10971) and 10 U/mL IL-2 (Miltenyi Biotec, #130-09-745). On days 5 and 8, 1*10^6^ cells were collected and stained with CD25-APC (Miltenyi Biotec, #130-101-435), CD69-PE (Miltenyi Biotec, #130-099-382), and Ki-67-FITC (Miltenyi Biotec, #130-100-289) for 30 min at room temperature. Then, cells were fixed and resuspended as previously described.

### Statistical analysis

All statistical analyses, including multiple t-tests, paired t-test and Wilcoxon test were analysed using GraphPad Prism V8.4.3. The normality of each data set was confirmed using the Shapiro-Wilk test. Data are mean ± SD as indicated.

## Supplementary Information

Below is the link to the electronic supplementary material.

Supplementary Material 1.

## Data Availability

The data that support the findings of this study are available from the correspondingauthor upon reasonable request.

## Abbreviations

PLWH: People living with HIV-1
CNS: Central nervous system
MPC: Mitochondrial pyruvate carrier
MPCi: Mitochondrial pyruvate carrier inhibitor
ART: Antiretroviral therapy
TCA: Tricarboxylic acid
ECAR: ExtraCellular acidification rates
OXPHOS: Oxidative phosphorylation
PPP: pentose phosphate pathways
FA: Fatty acid
FAO: Fatty acid oxidation
FAS: Fatty acid synthesis
CA: Cell-associated
HDAC: Histone deacetylase
LRA: Latency reversing agents
US RNA: Unspliced RNA
MS RNA: multi-spliced RNA
OCR: Oxygen consumption rates
SRC: Spare respiratory capacity
MRC: Maximal respiration capacity
GlycoATP: Glycolytic ATP
MitoATP: Mitochondrial ATP
ROS: Reactive oxygen species
LDH: Lactate dehydrogenase
PDE: Phosphodiesterase
α-KG: α-ketoglutarate
OAA: Oxaloacetate
S-CoA: Succinyl-CoA
Aa: Amino acid
LC: Lactyl group
SAM: S-Adenosyl methionine
