# Supplementary material for: Metabolic reprogramming of CD4⁺ T cells by Zaprinast induces HIV-1 latency reversal ex vivo
Source: Retrovirology. 2026 Jun 13;23:10. doi: 10.1186/s12977-026-00680-x (PMC13491861; doi:10.1186/s12977-026-00680-x)
Supplement: Supplementary file 1 [file 12977_2026_680_MOESM1_ESM.docx]

**Supplementary Table 1: List of reagents, kits, primers, and devices**

| **Name** | | **Manufacturer** | **Cat number (#)** | **Additional information** |  |  |
| --- | --- | --- | --- | --- | --- | --- |
| **Medium** | | | | |  |  |
| RPMI 1640 Medium, GlutaMAX™ Supplement | | Gibco | 61870010 |  |  |  |
| RPMI 1640 Medium | | Gibco | 11875101 |  |  |  |
| RPMI 1640 Medium, no glucose | | Gibco | 11879020 |  |  |  |
| DPBS, no calcium, no magnesium | | Gibco | 14190094 |  |  |  |
| Seahorse XF RPMI assay medium pack, pH 7.4 | | Agilent | 103680-100 |  |  |  |
| Seahorse XF 1.0 M Glucose solution | | Agilent | 103577-100 |  |  |  |
| Seahorse XF 200 mM L-Glutamine solution | | Agilent | 103579-100 |  |  |  |
| Penicillin-Streptomycin (10,000 U/mL) | | Gibco | 15140122 |  |  |  |
| Fetal Bovine Serum, qualified, heat inactivated. | | Gibco | 10500-064 |  |  |  |
| **Antibodies** | | | | |  |  |
| PerCP anti-human CD3 | | Biolegend | 300428 | 1:100; Clone:UCHT1 |  |  |
| CD4 Antibody, anti-human, FITC | | Miltenyi Biotec | 130-113-213 | 1:100; Clone:VIT4 |  |  |
| PE anti-human CD69 Antibody | | Biolegend | 310906 | 1:100; Clone FN50 |  |  |
| APC/Cyanine7 anti-human CD25 Antibody | | Biolegend | 302614 | 1:100; Clone:BC96 |  |  |
| Anti-Ki-67-FITC, human and mouse monoclonal | | Miltenyi Biotec | 130-100-289 | 1:100; Clone REA183 |  |  |
| CD45RA-PerCP | | Miltenyi Biotec | 130-095-466 | 5µL for 1*10^6^ cells, Clone: T6D11 |  |  |
| CD45R0-FITC | | Miltenyi Biotec | 130-095-462 | 5µL for 1*10^6^ cells, Clone:UCHL1 |  |  |
| CD25-APC | | Miltenyi Biotec | 130-101-435 | 5µL for 1*10^6^ cells, Clone:3G10 |  |  |
| CD69-PE | | Miltenyi Biotec | 130-099-382 | 5µL for 1*10^6^ cells |  |  |
| CD197-Vio770 | | Miltenyi Biotec | 130-099-164 | 5µL for 1*10^6^ cells |  |  |
| CD27-Vioblue | | Miltenyi Biotec | 130-099-502 | 5µL for 1*10^6^ cells, Clone:M-T271 |  |  |
| **Staining** | | | | |  |  |
| Viobility 405/452 Fixable Dye | | Miltenyi Biotec | 130-110-205 | 01:50 |  |  |
| MitoTracker™ Red CMXRos | | Invitrogen | M7512 | 100 nM |  |  |
| MitoView™ Green | | Generon | 70054 | 200 nM |  |  |
| MitoSOX™ Red Mitochondrial Superoxide Indicators | | Thermofisher | M36007 | 5 µM |  |  |
| 2-NBDG | | Invitrogen | N13195 | 50 µM |  |  |
| CellTrace™ CFSE Cell Proliferation Kit | | Invitrogen | C34554 | 5 µM |  |  |
| **Reagent** | | | | |  |  |
| DMSO | | Sigma-Aldrich | D4540 |  |  |  |
| Zaprinast | | Sigma-Aldrich | 684500-25MG | 4 µM – 16 µM |  |  |
| Phorbol 12-myristate 13-acetate (PMA) | | Merck | P8139-1MG | 20 nM |  |  |
| Ionomycin | | Sigma-Aldrich | I0634 | 280 nM |  |  |
| CD3/CD28 activator | | Stemcell Technologies | 10971 | 25 µL/mL |  |  |
| ImmunoCult™ Human CD3/CD28 T Cell Activator | | Stemcell Technologies | 10991 | 25 µL/mL |  |  |
| 2-Deoxy-D-glucose (2-DG) | | Sigma-Aldrich | D6134 | 50 mM |  |  |
| Antimycin A | | Sigma-Aldrich | A8674 | 1 µM |  |  |
| Oligomycin | | Merck | 75351 | 2.5 µM |  |  |
| Carbonyl cyanide 4-(trifluoromethoxy)phenylhydrazone (FCCP) | | Sigma-Aldrich | C2920 | 2 µM |  |  |
| Human recombinant IL-2 IS premium grade. | | Miltenyi Biotec | 130-09-745 | 10 U/mL |  |  |
| Ethylenediaminetetraacetic acid disodium salt solution (EDTA) | | Sigma-Aldrich | 03690-100mL | 1% |  |  |
| TNF-α human | | Sigma-Aldrich | SRP3177-50UG | 10 ng/mL |  |  |
| **Kit** | | | | |  |  |
| EasySep™ Direct Human CD4+ T Cell Isolation Kit | | Stemcell Technologies | 19662 | 50 (blood), or 55 (buffy coats) µL/mL |  |  |
| Rosette Sep CD8+ T cells depletion kit | | Stemcell Technologies | 15663 | 50 µL/mL |  |  |
| Ficoll Paque | | GE Healthcare | 17-1440-02 |  |  |  |
| BD Cytofix/Cytoperm fixation/permeabilization Kit | | BD Biosciences | 5547s14 | 100 µL/well |  |  |
| QIAamp Viral RNA Mini Kit | | Qiagen | 52904 |  |  |  |
| RNeasy Mini Kit | | Qiagen | 74106 |  |  |  |
| Verso one step rt-qPCR | | Thermo Scientific | AB-1454/LD/B |  |  |  |
| Quantinova probe qPCR | | Qiagen | 208252 |  |  |  |
| QuantiTect Probe RT-PCR | | Qiagen | 204445 |  |  |  |
| Taq DNA Polymerase | | Qiagen | 201203 |  |  |  |
| dNTP 100mM set | | Qiagen | 201912 |  |  |  |
| PCR grade water | | Thermofisher | R0582 |  |  |  |
| Proteinase K | | Fisher Scientific | AM2546 | 400 µg/mL |  |  |
| Seahorse XF Cell Mito Stress Test | | Agilent | 103010-100 |  |  |  |
| Glycolysis stress test | | Agilent | 103020-100 |  |  |  |
| Seahorse XF Real-Time ATP Rate Assay Kit | | Agilent | 103591-100 |  |  |  |
| pH-Xtra Glycolysis Assay | | Agilent | PH-200-4 |  |  |  |
| **HIV-1 p24 ELISA Assay** | | | | |  |  |
| 96-well Nunc maxisorp immune plate | | ThermoFisher | 442404 |  |  |  |
| Empigen 35% | | Sigma Aldrich | 45165 |  |  |  |
| P24 protein | | Aalto Bio Reagents | AG6054 |  |  |  |
| Anti p24 coating antibody | | Aalto Bio Reagents | D7320 |  |  |  |
| Anti p24 mAb biotinylated conjugate | | Aalto Bio Reagents | BC1071-Biot |  |  |  |
| Streptavidin | | Roche | 11-089-161-001 |  |  |  |
| Phosphatase substrate | | Sigma Aldrich | S0942 |  |  |  |
| **Primers** | | **Sequence 5’-3’** | |  |  |  |
| HIV-1 polyA tails | US-Forward | cagatcctgcatataagcagctg | | [88] |  |  |
|  | US-Reverse | ttttttttttttttttttttttttgaagcac | |  |  |  |
|  | US-Probe | **FAM**-cctgtactgggtctctctgg-**BHQ1** | |  |  |  |
| HIV-1 *gag* | HIV-1-Forward | tactgacgctctcgcacc | | [59] |  |  |
|  | HIV-1-Reverse | tctcgacgcaggactcg | |  |  |  |
|  | HIV-1-Probe | **HEX**-ctctctccttctagcctc-**BHQ1** | |  |  |  |
| HIV-1 *tatrev* | tat2 | acagtcagactcatcaagtttctctatcaaagca | | [90] |  |  |
|  | tat1.4 | tggcaggaagaagcggaga | |  |  |  |
|  | rev | ggatctgtctctgtctctctctccacc | |  |  |  |
|  |  |  |  |  |  |  |
|  | Probe HIV | **HEX**-ttccttcgggcctgtcgggtccc-**BHQ1** | |  |  |  |
| Housekeeping gene | TBP-Forward | cagtgaatcttggttgtaaacttga | | [91] |  |  |
|  | TBP-Reverse | tcgtggctctcttatcctcat | |  |  |  |
|  | TBP-Probe | **CY5**-cgcagcaaaccgcttgggattat-**BHQ2** | |  |  |  |
| RT-qPCR | US HIV-1 - Forward | gctgccaaagagtgatctga | |  |  |  |
|  | US HIV-1 - Reverse | tcttcagagcag’ccagagc | |  |  | |
|  | MS HIV-1 - Forward | tctatcaaagca’cccacct | |  |  | |
|  | MS HIV-1 - Reverse | gctaaggatccgttcactaa | |  |  | |
|  | YWHAZ - Forward | acttttggtacattgtggcttcaa | |  |  | |
|  | YWHAZ – Reverse | ccgccaggacaaaccagtat | |  |  |  |
| **Plastic** | | | | |  |  |
| 96-well Clear Round Bottom TC-treated Cell Culture Microplate | | Corning | 353077 |  |  |  |
| **Cell lines** | | | | |  |  |
| ACH-2 | | RRID: CVCL 0138; [80] | | |  |  |
| **Software & Devices** | | | | |  |  |
| Cytexpert | | Beckman Coulter - V2.4.0.28 | |  |  |  |
| Graphpad | | V8.4.3 | |  |  |  |
| SoftMax Pro | | Molecular Device - V7.1.2 | |  |  |  |
| Design&Analysis Software | | Thermofisher Scientific - V2.6.0 | |  |  |  |
| Seahorse Analytics | | Agilent - V1.0.0-699 | |  |  |  |
| Thermocycler 2720 | | Applied Biosystems | 272S7121870 |  |  |  |
| Quantstudio 5 | | Applied Biosystems | A28134 |  |  |  |
| Cytoflex | | Beckman Coulter | C02945 |  |  |  |
| Cytoflex S | | Beckman Coulter | B75408 |  |  |  |
| LinRegPCR | |  |  | [92] |  |  |

**Supplementary Figure 1: Gating strategy of CD25 and CD69 staining on CD4+ T cells from blood donors.** (one representative experiment). (a) Cells event selection and single cell gating (b) Isotype control for CD25 and CD69. (c), (d) Positive staining in DMSO and Zaprinast condition. (e) Positive staining in PMA-Ionomycin condition (positive control).

**Supplementary Figure 2: Zaprinast treatment moderately modulates mitochondrial membrane potential without inducing ROS production.**
(a) Normalised mitochondrial membrane potential relative to mitochondrial mass and DMSO treatment for each time point on CD4^+^ T cells from PBMC from blood donors. (n=4). Statistical analysis was performed using paired t-tests versus DMSO. (b) Mitochondrial Reactive Oxygen Species (ROS) production in CD4^+^ T cells measured immediately, 18-hours and 72-hours after treatment with DMSO or 4 µM Zaprinast (n=4). Statistical analysis was performed using paired t-tests versus DMSO.
